# Health-related quality of life of patients with diagnosed type 2 diabetes in Felege Hiwot Referral Hospital, North West Ethiopia: a cross-sectional study

**DOI:** 10.1186/s13104-018-3625-x

**Published:** 2018-08-02

**Authors:** Kidist Reba, Zeleke Argaw, Bizuayehu Walle, Hordofa Gutema

**Affiliations:** 1 Department of Adult Health Nursing, School of Health Science, College of Medicine and Health Science, Bahir Dar University, Bahir Dar, Ethiopia; 2 College of Medicine, Addis Ababa University, Addis Ababa, Ethiopia; 3 Department of Physiology, School of Medicine, College of Medicine and Health Science, Bahir Dar University, Bahir Dar, Ethiopia; 4 Department of Health Promotion and Behavioral Sciences, School of Public Health, College of Medicine and Health Science, Bahir Dar University, Bahir Dar, Ethiopia

**Keywords:** Health-related quality of life, Type 2 diabetes, WHOQOL-BREF

## Abstract

**Objective:**

Diabetes mellitus is a chronic non-communicable disease with considerable impact on health status and quality of life. It has a profound effect on quality of life in terms of social and psychological as well as physical well-being. This study was conducted to assess health related quality of life among patients with diagnosed type 2 diabetes.

**Result:**

A cross-sectional study design was conducted from April to May, 2015. World Health Organization quality of life-BREF tool was used for collecting the data. A total of 344 patients with diagnosed type 2 diabetes were involved in the study. The overall health related quality of life mean score of the study participants was 52.6 ± 12.1 SD. Social domain has higher mean score (57.8 ± 14.8 SD). Educational status, marital status, occupation, duration of the diabetes and diabetes related complications had statistically significant association with health-related quality of life. An intervention that give special attention to the breaking of the cycle of low occupational status and literacy; and which encourage patients with type 2 DM to have good control of their diabetes and prevent complication should be implemented to improve their quality of life.

## Introduction

Diabetes mellitus (DM) is defined as a chronic disease that occurs either when the pancreas fails to produce sufficient insulin which is type 1 diabetes or when the body cannot effectively use the produced insulin which is called type 2 diabetes. If it is not controlled, diabetes will result with hyperglycemia or raised blood sugar and over time leads to serious damage to the body’s systems, particularly the nerves and blood vessels [1, 2].

Quality of life (QOL) is defined as individuals’ perceptions of their position in life in the context of the culture and value systems in which they live and in relation to their goals, expectations, standards and concerns [3]. It is a known fact that DM is associated with increased morbidity and mortality globally. However, its effect on functional health status and sense of wellbeing is still not well addressed, particularly in developing country where the prevalence is rising rapidly [2].

Different scholars have conducted study on QOL of patients diagnosed with diabetes and had reported that diabetes has a negative impact on HRQOL. However, since the tool used by these researchers to measure HRQOL was different among the studies and some of the studies have used small sample size, it is still arguable to know the correct finding about the QOL among patient with diagnosed type 2 diabetes [4–8]. Contrary to rapid increase of type 2 diabetes prevalence, studies conducted in developing countries are few in number and majority of them focused on both type of the diabetes, while those studies conducted on type 2 diabetes were done among patients who have experienced only specific DM complication [4, 6, 9, 10].

In Ethiopia, there is an increment in the trend of diabetes prevalence since 1980 with currently prevalence of 3.8%. It also accounts 1% of total deaths of all age in the country. According to International Diabetes Federation (IDF), estimation, the 2.5 prevalence of diabetes in 2011 will rise to 3.5% by the year 2030 [2, 11]. To combat the burden of this diabetes and other non-communicable disease (NCD), the country has implemented a national strategy based on the available evidence [12]. Since there is no study conducted on quality of life of patient with diagnosed DM to our knowledge, the current study will fill the existing gap in quality of life the patients.

Indeed, studies have been conducted on DM in Ethiopia. But, to our knowledge, all of them were undertaken to assess prevalence of DM and factors associated with it and there is no any study which conducted on HRQOL, specifically on type 2 diabetes [13–15]. Hence this study was conducted by aiming to assess HRQOL and its associated factors among type 2 diabetic patients on follow up in Felege Hiwot Referral Hospital (FHRH), North West Ethiopia.

## Main text

### Study design and setting

A cross-sectional study was employed from April 1 to May 15, 2015. The study was conducted among patients with diagnosed type 2 diabetes at FHRH which is found in Bahir Dar city. Bahir Dar city which is located on northwest and 565 km away from Addis Ababa; the capital of Ethiopia. FHRH is the only governmental hospital in the city. It has 200 beds and three medical OPD serves for medical patients of which one serve as referral and follow up clinic for patients with chronic diseases. Patients diagnosed with DM constitute larger number among patients attending in the follow up clinic.

### Sample size and Sampling

Patients diagnosed with type 2 diabetes who had follow up for ≥ 1 year, aged > 18 years and visited the facility for follow up during the study period were selected and participated in this study after excluding patients who had history of substance abuse such as alcohol and cigarette.

### Sample size and sampling procedure

Sample size was calculated using a single population proportion formula assuming proportion of HRQOL among 2 DM patients 50%, 5% margin of error (d) and 95% (zα/2 = 1.96) and thus, the final sample size was calculated to be 384.

A systematic sampling technique was employed to select study participants. After developing the sampling frame from the FHRH’s follow up register, the sampling interval was calculated by dividing the number of individuals in the frame by the sample size.

### Data collection procedure

Data was collected using interviewer administered structured questionnaire. The questionnaire adapted from validated instrument of WHOQOL-BREF tool [3]. The English version questionnaire was translated to Amharic (local language) and back translated to English by other person to check the consistence of the meaning. Four data collectors and one supervisor who are health professional were recruited.

### Instruments

The instrument comprised of socio-demographic characteristics, DM related questions and the WHO-QOL tool. The WHOQOL-BREF is a 26-item instrument consisting of four domains: physical health (7 items), psychological health (6 items), social relationships (3 items), and environmental health (8 items), overall perception of QOL and general health (2 items). Each item of the WHOQOL-BREF is scored from 1 to 5 on a response scale, which is stipulated as a five-point ordinal scale. Three negatively worded items were reversely coded before attempt of any analysis. The score of each domain was obtained by summation of their corresponding items. The scores were then transformed linearly to a 0–100-scale. Participants were weighed in kg in light clothing and bare feet. Height was measured using a measuring tape; participants stood in erect posture without shoes, and was recorded to the closer 0.5 cm. Body mass index (BMI) was calculated as the ratio of weight in kilograms to the square of height in meters.

### Data analysis

Data were entered into Epi-Data version 3.1 and exported to statistical package for social Sciences (SPSS) version 20 for analysis. All variables having a p value of ≤ 0.2 in the bivariate analysis were further entered in to multi variable regression. Backward step wise linear regression was performed. The result of the β-coefficient was used for interpretation of strength of prediction of the independent variables to the HRQOL. For all statistical significance tests, the cut- off value set was p < 0.05 at Confidence interval of 95%.

## Results

### Socio-demographic characteristics

Making the response rate 90%, a total of 344 patients with diagnosed type 2 diabetes were participated in the study. More than half (57.3%) of the participant were male. The mean age of the participants was 40.5 ± 15.2 years. As of marital status, majority (60.8%) of the participants were married. Regarding the educational status, 143 (41.6%) of them completed secondary school and above (Table 1).

**Table 1:** Socio-demographic characteristics of patients with diagnosed type 2 diabetes attending FHRH, North West Ethiopia 2015

Variable	Frequency (N)	Percentage (%)
Sex		
Male	197	57.3
Female	147	42.7
Age (years)	Mean and SD (40.54 ± 15.208)	
Educational status		
Can’t read and write	117	34
Read and write but no formal educ.	23	6.7
Primary education	61	17.7
Secondary education and above	143	41.6
Religion		
Orthodox	267	77.6
Muslim	70	20.3
Protestant	7	2.1
Ethnicity		
Amhara	336	97.6
Gurage	4	1.2
Tigre	4	1.2
Marital status		
Single	62	18
Married	209	60.8
Divorced	33	9.6
Widowed	40	11.6
Occupation		
Government employee	104	30.2
Unemployed	77	22.4
Merchant	48	14.0
Student	22	6.4
Farmer	76	22.1
Private employee	17	4.9
Monthly income (in birr)	Mean and SD (1962.6 ± 6.5)	

### Medical history and health condition

As presented on Table 2, more than half (57%) of the study participants were living with DM for less than 5 years and only 10 (2.9%) of them living with it for more than 15 years. One third (64.3%) of the participants are currently using insulin for the DM. Above half (53.5%) of them have experienced DM complication. Of those who experienced DM complication, one fourth (25%) of them have developed Diabetic retinopathy and 40 (21.7%) of them experienced two or more complication. Regarding the body mass index, majority (58.7%) of them found in normal range (18.5–25) of BMI (Table 2).

**Table 2:** Medical history and health condition of patients with diagnosed type 2 diabetes attending FHRH, North West Ethiopia 2015

Variable	Frequency (N)	Percent (%)
Duration of DM (years)		
≤ 5	196	57
6–10	71	20.9
11–15	66	19.2
> 15	10	2.9
Drug regimen		
Oral anti DM medication	74	21.5
Insulin	221	64.3
Insulin and oral anti DM medication	49	14.2
Presence of diabetes complication		
Yes	184	53.5
No	160	46.5
Type of complication (n = 184)		
Diabetic nephropathy	21	11.4
Diabetic neuropathy	14	7.6
Diabetic retinopathy	47	25.6
Diabetic foot ulcer	39	21.2
Diabetic related heart disease	23	12.5
Two and above complication	40	21.7
Body mass index (kg/m^2^)		
< 18.5	16	4.7
18.5–25	202	58.7
25–30	105	30.5
> 30	21	6.1

### Factors associated with overall HRQOL

As presented in Table 3, Patients with diagnosed type 2 diabetes who are able to read and write and completed secondary school and above have 5.16 and 7.3 more HRQOL score than those who can’t read and write (β = 5.16, p-value = 0.019] and (β = 7.3, p-value = 0.000) respectively. Patients who are divorced and widowed have − 7.35 and − 4.65 less HRQOL score than patients who are single (β = − 7.35, p-value = 0.001) and (β = − 4.65, p-value = 0.033) respectively. Merchant and private employee patients who are living with diagnosed type 2 DM have 5.1 and 7.6 more HRQOL score than government employee (β = 5.09, p-value = 0.003) and (β = 7.60, p-value = 0.007) respectively, while unemployed patients have − 4.5 less HRQOL score than government employee patients (β = − 4.05, p-value = 0.015).

**Table 3:** Factors associated with overall HRQOL among patients with diagnosed type 2 diabetes attending FHRH, North West Ethiopia, 2015

Variables	Beta	Overall HRQOL		
		p	95% CI	
			Lower	Upper
Age	− 0.03	0.536	− 0.12	0.06
Income (in Birr)	0.0	0.683	− 0.001	0.00
Educational status				
Can’t read and write	1	1	1	1
Read and write	5.16	0.019*	0.85	9.46
Primary school	0.52	0.758	− 2.78	3.84
Secondary school and above	7.3	0.000*	4.00	10.62
Marital status				
Single	1	1	1	
Married	2.01	0.195	− 1.03	5.05
Divorced	− 7.35	0.001*	− 11.64	− 3.06
Widowed	− 4.65	0.033*	− 8.94	− 0.37
Occupation				
Government employee	1	1	1	1
Unemployed	− 4.05	0.015*	− 7.32	− 0.78
Merchant	5.09	0.003*	1.71	8.47
Student	− 1.20	0.636	− 6.19	3.79
Farmer	1.25	0.493	− 4.83	2.33
Private employee	7.60	0.007*	2.05	13.13
Duration of DM (years)				
≤ 5	1	1	1	1
6–10	− 4.09	0.009*	− 7.14	− 1.05
11–15	− 6.48	0.000*	− 9.40	− 3.55
> 15	2.41	0.478	− 4.27	9.01
Drug regimen				
Oral anti DM medication	1	1	1	1
Insulin	− 1.73	0.226	− 4.53	1.07
Insulin and oral DM medication	− 2.38	0.194	− 5.96	1.22
DM complication				
No	1	1	1	1
Yes	− 4.82	0.000*	− 7.23	− 2.42
Body mass index (kg/m^2^)				
< 18.5	1	1	1	1
18.5–25	− 0.47	0.86	− 5.77	4.83
25–30	− 1.34	0.63	− 6.80	4.13
> 30	− 5.78	0.85	− 12.41	0.81

* Significant at p < 0.05

Patients who are living with diagnosed type 2 diabetes for 6–10 and 11–15 years have − 4.1 and − 6.48 less HRQOL score than those who are living with it for 5 years and below (β = − 4.09, p-value = 0.009) and (β = − 6.48, p-value = 0.000) respectively. Patients who experienced any of DM complication have − 4.82 less HRQOL score than those who didn’t develop any DM complication (β = − 4.82, p-value = 0.000) (Table 3).

## Discussion

The current study demonstrated the HRQOL of patients living with diagnosed type 2 diabetes and attending DM follow up clinic in FHRH. In this study, we are able to identify 52.6 ± 12.1 mean score of the overall HRQOL among patient with diagnosed type 2 diabetes. This finding is comparable with the previous studies which used the WHOQOL-BREF scale to determine HRQOL of patients with DM. In studies conducted in Iran and Canada, the mean score of overall QOL was 51.2 and 54.7 in patient with diagnosed type 2 diabetes [14, 15]. However, this finding is lower than that of study among Emirate people with DM which showed 63.1 mean score of overall HRQOL [16].

Among the four domains of HRQOL, study participants had the higher mean score (57.8) on social domains and lower mean score (48.1) in physical domain. Although they have different figure, this highest mean score in social domain was also reported in study among Iranian, Nigerian, Kenyan, Mexican, refugee in Gaza and Emirate people with DM [9, 14, 16–19]. Individual with type 2 DM who are able to read and write, completed high school and above have better HRQOL than those who have no education. The finding of the studies conducted in Iran and Mexico on QOL also showed that HRQOL is significantly associated with educational status of patients with type 2 DM [8, 16, 20], implying that patients with type 2 DM who have higher educational status have better ability to make decision on self-care, better understanding about the disease, its complication and treatment.

Divorced and widowed patients with type 2 DM have lower QOL than those who are single. This relationship between marital status and QOL is also observed in previous studies conducted on DM patients [8, 16, 18, 19]. HRQOL of unemployed patients with type 2 DM is lower as compared with those patients who are government employee, private employee and merchants. A national survey conducted in Iran has also reported significant association between employment and HRQOL of patients with type 2 DM [8]. A study done in Nigeria on QOL of patients with DM has also showed significant association of occupation with QOL [6].

Patients who are living with type 2 DM for more than 5 years have worse HRQOL as compared with those who live with it for 5 years and less. This negative relationship between QOL and duration of DM is demonstrated in previous studies. In study conducted among Iranian and Emirates patients with diabetes has also shown the influence that duration of DM has on the QOL of patients [8, 18]. The current study has also observed significant association between complication of DM and HRQOL of patients. It has demonstrated that patients with type 2 DM who have developed any DM complication have worse QOL. The negative effect of DM complication on HRQOL of patients with DM has been reported in previous studies [5, 6, 18].

## Conclusion

The finding of the current study showed low mean score of overall and domain of HRQOL. The HRQOL of patients diagnosed with type 2 DM is affected by their educational status, marital status, occupation, duration of DM and presence of DM complication. An intervention that give special attention to breaking the cycle of low occupational status and literacy should be implemented to improve the quality of life of DM patients. Moreover, it could direct researchers to understand how each domain of HRQOL are being affected.

## Limitations

The limitations of the present study are; (1) the utilization of cross-sectional design; which lack reporting of causal relationship of the variables. (2) It has to be noted that the data was collected using interviewer administered questionnaire which may make the finding prone to social desirability bias.

## Abbreviations

BMI: body mass index
DM: diabetes mellitus
FHRH: Felege Hiwot Referral Hospital
FMOH: Federal Ministry of Health
HRQOL: health related quality of life
IDF: International Diabetic Federation
NCD: non-communicable disease
OPD: out patient department
QOL: quality of life
SD: standard deviation
SPSS: statistical package for social sciences
WHO: World Health Organization
WHO-QOL: World Health Organization quality of life
